# Genomic Signals of Adaptation towards Mutualism and Sociality in Two Ambrosia Beetle Complexes

**DOI:** 10.3390/life9010002

**Published:** 2018-12-22

**Authors:** Jazmín Blaz, Josué Barrera-Redondo, Mirna Vázquez-Rosas-Landa, Anahí Canedo-Téxon, Eneas Aguirre von Wobeser, Daniel Carrillo, Richard Stouthamer, Akif Eskalen, Emanuel Villafán, Alexandro Alonso-Sánchez, Araceli Lamelas, Luis Arturo Ibarra-Juarez, Claudia Anahí Pérez-Torres, Enrique Ibarra-Laclette

**Affiliations:** 1 Red de Estudios Moleculares Avanzados, Instituto de Ecología A.C, Xalapa, Veracruz 91070, Mexico; jazminblax@gmail.com (J.B.); mirnavrl@gmail.com (M.V.-R.-L.); anahi.canedo@posgrado.ecologia.edu.mx (A.C.-T.); emanuel.villafan@inecol.mx (E.V.); alexandro.alonso@inecol.mx (A.A.-S.); araceli.lamelas@inecol.mx (A.L.); luis.ibarra@inecol.mx (L.A.I.-J.); claudia.perez@inecol.mx (C.A.P.-T.); 2 Departamento de Ecología Evolutiva, Instituto de Ecología, Universidad Nacional Autónoma de México, Ciudad de México 04500, Mexico; josue_barrera@comunidad.unam.mx; 3 Cátedras CONACyT/Centro de Investigación en Alimentación y Desarrollo, Hermosillo 83304, Mexico; eneas.aguirre@ciad.mx; 4 Tropical Research and Education Center, University of Florida, Homestead, FL 33031, USA; dancar@ufl.edu; 5 Department of Plant Pathology, University of California–Riverside, Riverside, CA 92521, USA; richards@ucr.edu; 6 Department of Plant Pathology, University of California, Davis, CA 95616-8751, USA; aeskalen@ucdavis.edu; 7 Cátedras CONACyT/Instituto de Ecología A.C., Xalapa, Veracruz 91070, Mexico

**Keywords:** mutualism, sociality evolution, ambrosia beetle complexes, polyphagous shot hole borer, redbay ambrosia beetle

## Abstract

Mutualistic symbiosis and eusociality have developed through gradual evolutionary processes at different times in specific lineages. Like some species of termites and ants, ambrosia beetles have independently evolved a mutualistic nutritional symbiosis with fungi, which has been associated with the evolution of complex social behaviors in some members of this group. We sequenced the transcriptomes of two ambrosia complexes (*Euwallacea* sp. near *fornicatus*–*Fusarium euwallaceae* and *Xyleborus glabratus–Raffaelea lauricola*) to find evolutionary signatures associated with mutualism and behavior evolution. We identified signatures of positive selection in genes related to nutrient homeostasis; regulation of gene expression; development and function of the nervous system, which may be involved in diet specialization; behavioral changes; and social evolution in this lineage. Finally, we found convergent changes in evolutionary rates of proteins across lineages with phylogenetically independent origins of sociality and mutualism, suggesting a constrained evolution of conserved genes in social species, and an evolutionary rate acceleration related to changes in selective pressures in mutualistic lineages.

## 1. Introduction

By definition, mutualistic symbiosis increases the fitness of all participant partners, at least in terms of inclusive fitness [1]. Therefore, mutualistic lineages are interdependent, and natural selection drives their coevolution [2]. Elucidating how these mutualistic partnerships influence genes and genomes is essential to understanding complex ecological interactions in an evolutionary context, and is thus a fundamental aim of life sciences.

One of the most notable cases of mutualistic symbiosis in nature is the farming of fungi by several insect groups [2]. Nutritional mutualistic symbiosis with fungi has evolved over tens of millions of years in Attini ants, Macrotermitinae termites, and Scolytinae or Platypodinae ambrosia beetles, enabling these organisms to colonize new ecological niches [1,2]. Of these fungus-associated lineages, ambrosia beetles are probably the least studied, and constitute an interesting model system for studying the evolutionary transition to an obligate mutualism in insects.

Ambrosia beetles comprise a polyphyletic species assemblage that includes some members of the Scolytinae and Platypodinae subfamilies, in which fungus farming has evolved independently at least 12 times as the dominant ecological strategy [3,4]. Tracking physiological and behavioral traits indicates coadaptation to a mutualistic lifestyle with fungi. For instance, they have evolved specialized integument structures called mycangia, where fungal spores are transported for their cultivation on the walls of intricate gallery systems bored into the xylem of host trees [5,6,7]. Moreover, the presence of the beetles can trigger physiological responses from the fungus, including the development of fruiting bodies [8], indicating sophisticated inter-species regulatory processes, which are likely the result of intensive long-term coevolution. 

Caring for the fungal gardens involves cooperative behavior, and could be related to the decrease of inter- and intra-specific competition for food [5,9,10]. It has been proposed that these factors have promoted, through gradual evolutionary processes, the development of a facultative eusocial system in some lineages of ambrosia beetles [11]. While full eusocial behavior in ants and termites includes a division of individuals into groups called castes, with most individuals being unable to reproduce, the social structure observed in some scolytine beetles is less specialized [9,10,11]. This facultative eusociality is characterized by the overlapping of generations, parental care, division of labor between adults and larvae (age polyethism), and cooperative brood care, as has been described in *Xyleborus affinis* and *Xyleborinus saxeneii* [12,13,14].

Although, strictly speaking, this facultative eusociality has only been described in two species of the Xyleborini tribe [13,15,16], several factors suggest that high levels of sociality are conserved within this monophyletic group. The species of this tribe are haplodiploid, and typically mate among siblings within their natal brood chamber [17,18,19]. Their high genetic relatedness due to inbreeding and the high costs of dispersal potentially benefits the evolution of cooperative behaviors within the natal gallery (e.g., by fungus gardening, gallery extension, offspring feeding, and cleaning) [20], and could favor the evolution of facultative eusociality. 

There are two general and not mutually exclusive hypotheses that explain the molecular mechanisms underlying the evolutionary transition from solitary living to sociality in insects, one based on changes in gene regulation and another based on protein sequence evolution [21,22,23]. The former, known as the ‘genetic toolkit’ hypothesis, proposes that deeply conserved genes and gene networks with roles in solitary behaviors are co-opted through changes in gene regulation, leading to the evolution of social traits such as social foraging, reproductive dominance, and age polyethism [21,22,23]. The second hypothesis postulates that a wide diversity of behaviors and phenotypes arose through the expansion, neofunctionalization, and selection of lineage-specific gene families involved in functions such as carbohydrate metabolism, glandular development, and signal transduction [21,22,23]. Research supports both general hypotheses, and shows highly conserved genes affecting the expression of complete networks that are caste-biased and influence social traits [24,25,26,27], and a small overlap of genes associated with social behavior among distant lineages [26,28,29,30]. Moreover, role division in animal social groups implies traits which increase the fitness of other members of the group, often at the expense of the individuals harboring the trait. In alloparental care, for example, individuals care for their siblings, instead of devoting those efforts to producing their own brood [6]. These altruistic behaviors can be explained through kin selection and inclusive fitness [31,32], where caring for related individuals increases the overall fitness of the related genotype, as it is shared between individuals in some proportion. Therefore, natural selection through inclusive fitness should play a role in the evolution of sociality.

In this context, recent comparative genomic research has identified genomic signals associated with social evolution in ants and termites. Expansion and positive selection of gene families involved in the production and perception of pheromones, caste determination, molting, and metamorphosis has been documented for termites [33]. Meanwhile, Attine ant genomes show very high rates of structural rearrangement [34] and changes in the regulation of gene expression between castes, which could be associated with the rate of evolution of genes with specific caste profiles, as well as genes coding transcriptional regulators [35]. Changes in the molecular evolutionary rate have also been found in organisms with mutualistic lifestyles, such as an accelerated substitution rate in lichen species [36] and increased rates of genome evolution in the *Pseudomyrmex* ants that form mutualistic rather than generalist relationships with plants of the genera *Acacia*, *Triplaris*, and *Tachigali* [37].

In order to identify genomic signals associated with the evolution of obligate mutualism and putative facultative eusociality in ambrosia beetles, we performed an evolutionary analysis based on the transcriptome sequencing of two ambrosia complexes (beetle and fungi): the polyphagous shot hole borer (PSHB), *Euwallacea* sp. near *fornicatus*–*Fusarium euwallaceae* S. Freeman, Z. Mendel, T. Aoki & O’Donnell [38,39] and Redbay ambrosia beetle (RAB) *Xyleborus glabratus* Eichhoff, 1877–*Raffaelea lauricola* T.C. Harr., Fraedrich & Aghayeva 2008 [40]. These two complexes belong to the monophyletic Xyleborini tribe, and have been recently described as very hazardous pests for forest health, landscape trees, and the avocado industry, being the causal agents of the diseases commonly known as *Fusarium* dieback and laurel wilt, respectively [5,39,41,42,43,44].

We jointly sequenced the transcriptome of the beetles and the fungi present in their bodies. We performed a screening to separate the fungus-like sequences from the beetle sequences, to assess their functions and their relation to the establishment of their mutualistic interactions. 

Finally, we performed a comparative analysis between the transcriptomic data of these two ambrosia beetles and the genomes of other insects. The species considered in the comparative analysis exhibit a wide range of social structures, from solitary to eusocial, and represent four independent origins of sociality (termites, bees, ants, and wasps). Moreover, we included an independent origin of fungus farming mutualism by adding the genomes of four Attini ant species to the comparative analysis. Through this approach, we identified genes that have been selected during the evolution of obligate mutualism and sociality in ambrosia beetles; we further evaluated the relationship between the molecular evolutionary rate and both sociality and obligate mutualism in insects that have convergently evolved these traits. 

## 2. Materials and Methods

### 2.1. Beetle Collection 

*Xyleborus glabratus* beetles were collected from silk bay (*Persea humilis* Nash) trees from Highlands County, Florida showing laurel wilt symptoms, including wilted foliage and strings of boring dust from numerous small holes. Visibly infested branches with diameters larger than 3 cm were placed in an insect emergence chamber to allow the beetles to emerge from the galleries. Once emerged, the beetles were sorted, identified, and stored in a commercial RNA-stabilizing buffer (RNAlater; Ambion) until RNA isolation. Beetle collection and identification were performed as described previously by Johnson et al. 2018 and Hulcr et al. 2017 [4,5]. Due to the low presence of *X. glabratus* among the collected beetles, around 5–7 live adult females were used for total RNA isolation. 

### 2.2. Reared Beetles

*Euwallacea* sp. near *fornicatus* beetles were harvested from established colonies maintained at the University of California Riverside (UCR) Insectary and Quarantine facility. These colonies were initially established from live adult females, collected from avocado wood showing *Fusarium* dieback disease symptoms. Insects were raised on a semi-artificial diet based on the modified recipe of Biedermann et al. 2009 [12]. The diet was comprised of 1.5 g Wesson’s salt, 6 g sucrose, 6 g yeast extract, 6 g potato starch, 12 g casein, 35 g agar, 236 g castor bean sawdust and 685 mL water. The ingredients were mixed together inside a plastic tub before being packed into 15 mL Falcon centrifuge tubes. Tubes containing the diet were autoclaved at 120 °C for 40 min and allowed to rest for two days before use, to allow the evaporation of excess moisture. A single adult mated female was placed inside each tube, which was sealed with cotton. Beetles could form galleries within the media at 25 °C and ambient humidity. Every six weeks, female adults were then harvested from colony tubes by removing the media from the tube and dissecting them. A total of 60 live adult females were used for RNA isolation.

### 2.3. RNA Isolation, Library Preparation and Sequencing

Total RNA was isolated with TRIzol reagent (Life Technologies, Carlsbad, CA, USA) and purified with the RNeasy MiniElute kit (QIAGEN, Venlo, The Netherlands) according to the manufacturer’s instructions. In the case of *E.* nr. *fornicatus*, around 50% of the collected beetles were dissected to independently extract the total RNA from two sections: head-thorax and abdomen. The remaining insects were used to obtain RNA from the whole body. In the case of *X. glabratus*, RNA was isolated from the whole body only, due to the limited number of beetles collected. RNA integrity was assessed by chip-based capillary gel electrophoresis using an Agilent 2100 Bioanalyzer system (Agilent Technologies, Santa Clara, CA, USA). The RNA concentration was determined by absorbance at 260 nm using a NanoDrop 2000 UV-Vis spectrophotometer (Thermo Fisher Scientific, Waltham, MA, USA). A total of 500 ng RNA was used as input material to prepare each of the four RNA-seq libraries used in this study. We used the RNA-seq method based on poly(A) selection, which enriches for eukaryotic mRNA and other polyadenylated RNA molecules. This method is the most common protocol used in whole transcriptome sequencing projects because it provides a broad, detailed, and accurate view of the transcriptional landscape of the protein-coding genes. The TruSeq RNA Sample Preparation Kit (Illumina, San Diego, CA, USA) was used for this purpose, adding specific indexes to each of the sequenced samples. All libraries were sequenced on a NextSeq 550 platform (Illumina) using a 2 × 150 bp paired-end sequencing protocol, whereby 150 bases from each side of the DNA strands were sequenced.

### 2.4. Data Processing, Assembly, and Functional Annotation

Prior to the assembly process, low-quality reads were removed from the analysis according to previously defined criteria (Table S1). Paired-end reads with an average Phred quality score lower than 30, and in which at least 90% of the bases along the sequence failed to meet a Phred quality score of 20 or greater, were filtered out. A python-based script was used for this purpose (https://github.com/Czh3/NGSTools/blob/master/qualityControl.py).

Considering that in ambrosia beetle complexes, both insects and fungi are eukaryotic organisms, and fungi spores are contained inside the mycangia of the beetles, sequences from both (fungi and insect) were expected to be found in the sequenced libraries, although in different proportions. Therefore, before de novo assembly, screening was performed to identify fungus-like sequences, using the Hisat2 2.1.0 program [45]. Reference sequences used consisted on whole genome sequences and/or unique-transcript collections resulting from transcriptomic studies, available in public databases such as JGI (https://genome.jgi.doe.gov/portal/ [46,47]) and GeneBank (https://www.ncbi.nlm.nih.gov/). Only available sequences from fungi associated with ambrosia beetles were included [48,49,50] (see Table S2 for more details, [48,49,50,51,52,53])

The sequences were divided into four datasets based on the process described above: *X. glabratus* sequences, fungus-like sequences obtained from *X. glabratus*, *E.* nr. *fornicatus*, and fungus-like sequences obtained from *E.* nr. *fornicatus.* High-quality paired-end reads from each of these four datasets were independently assembled using Trinity 2.4.0 software [54] (Table S3), producing expressed sequences, which can be called transcript unigenes [55]. Before the annotation process, unigenes were processed with AlignWise [56], a pipeline which drives several programs such as BLAST [57], Muscle [58], and GeneWise [59] to identify and correct out-of-frame insertions/deletions in coding regions through a homology-based method. We created a database containing coding sequences and their corresponding proteins from 10 beetle genomes and 11 from other insects for homology-based correction of beetle transcriptomes (Table S4: A1–A3; [33,34,60,61,62,63,64,65,66,67,68,69,70,71,72,73]); the coding sequence and protein sequences of the fungal reference database (Table S2) were used to perform this step for fungus-like sequences.

After correcting the frame-shifts, redundant sequences were eliminated with BlastClust (https://www.ncbi.nlm.nih.gov/Web/Newsltr/Spring04/blastlab.html). A sequence was considered redundant if it showed an identity of at least 95% over 90% or more of the length of the sequences being compared. Only sequences longer than 30 amino acids were kept for further analyses.

To further reduce the presence of contaminating sequences in the two beetle-sequence datasets, we searched for fungus-like sequences that could have been missed in the first screening. This time, considering that distinct fungal species could be associated with the ambrosia beetles, the search was conducted among the unigenes obtained after the assembly and sequences-correction processes. BLASTp similarity searches (e-value ≤ 10^−10^) were independently performed against two distinct databases, one containing the proteins codified in the whole genome of 811 fungal species obtained from the ENSEMBLFungi genome browser (https://fungi.ensembl.org/species.html), and the other with the proteins codified in the genome of 12 distinct insect species (Table S4: A2). The unigenes with putative homologs identified in both databases were sorted based on bit-score value (from highest to lowest), and were treated as fungal sequences if they had a higher bit-score for the fungal database than for the insects. Other stringency parameters such as the identity and the coverage between the unigenes and their homologs were also considered (identity had to be at least 90% and coverage at least 70%).

The annotation process for the four generated transcriptomes included a functional classification and a search for similar sequences in sets of identified proteins in the genome sequences of some species, considered as references mainly due to the quality of their genome annotation and/or the phylogenetic relationships. Functional annotation was carried out by InterproScan5 analysis [74,75] using the applications Pfam [76,77], TIGRFAM [78], PIRSF [79], and SUPERFAMILY [80]. Functional categories were inferred from gene ontology (GO) information [70]. Furthermore, BLASTp analyses of beetle transcriptomes were conducted using *Dendroctonus ponderosae*, *Drosophila melanogaster*, and *Apis mellifera* as reference species. Moreover, we used *Raffaelea lauricola* and *Fusarium euwallaceae* proteomes in the fungus-like sequence, searching (BLASTp) and Gene Ontology (GO) terms enrichment analysis. These proteomes were obtained through the gene model prediction in previously sequenced genomes ([48,49]; Table S2), which are available through the NCBI database. *Ab initio* and evidence-direct predictor AUGUSTUS [81,82] was used to generate these first versions of the gene models, which were corrected and improved by MAKER pipeline [83,84], entering the whole genome sequences with masked transposable elements, the available transcriptional dataset [49,85], and a reference protein database containing complete proteomes from some ascomycetes available from the JGI database.

### 2.5. Data Availability 

Raw transcriptome data of the beetle transcriptomes are available from the NCBI Sequence Read Archive under the accession number PRJNA495609. 

### 2.6. Ortholog Group Identification

We identified ortholog genes among *X. glabratus*, *E.* nr. *fornicatus*, and other beetle species (*Dendroctonus ponderosae*, *Leptinotarsa decemlineata*, *Tribolium castaneum*, and *Oryctes borbonicus*) to gain insight into the selection acting on molecular traits related to the obligate mutualism, as well as to the facultative eusocial behavior displayed by these fungus-farming beetle species. The comparison also included the genomes of other insects with a variety of forms of social organization (*Apis mellifera*, *Polistes dominula*, *Cerapachys birori*, *Zootermopsis nevadensis*, and *Cryptotermes secundus)*, solitary lifestyles (*Frankliniella occidentalis)*, and fungus-farming mutualistic behaviors (*Atta cephalotes*, *Acromyrmex echinatior*, *Trachymyrmex zeteki*, and *Cyphomyrmex costatus*) (Table S4: A3). 

To identify and distinguish homologs and orthologs across compared species, we used the get_homologues.pl script [86,87]. GET_HOMOLOGUES is a versatile software package which combines tools such as OrthoMCL [88] and BLAST [57]. OrthoMCL is an analysis pipeline that uses reciprocal BLAST and the Markov cluster (MCL) algorithm [89] to infer and group orthologs (and paralogs) across multiple taxa. Gene families from predicted proteins were identified using a default MCL inflation value of 1.5, as well as a threshold value of 10^−10^, and a minimum percent of coverage of 75 in the BLAST step. Only those families with a single-copy gene per species were used for phylogenetic inference and evolution rate calculation (see below).

### 2.7. Evolutionary Analysis

We considered all gene families present in 3 or more species for downstream analyses. Multiple sequence alignments were obtained for each gene family, using amino acid sequences with MAFFT [90]. The amino acid alignments were used to obtain codon-based alignments using PAL2NAL v14 [91]. Unigenes with inconsistencies between peptide and nucleotide sequences were discarded from further analysis. Nucleotide alignments were used to construct approximate maximum likelihood (ML) phylogenies with FastTree Version 2.1.9 [92], using the generalized time-reversible evolution model and the Shimodaira-Hasegawa test for branch support calculation. 

Characterization of selective processes based on non-synonymous (dN) versus synonymous (dS) rates of protein-coding sequences was performed using the HyPhy v2.2 package (https://veg.github.io/hyphy-site/). FEL-contrast (fixed effect likelihood; [93]) was used to identify individual codons with significant changes in dN/dS ratio associated with the adaptation to the mutualistic ecological trait. In order to find lineages which have experienced natural selection pressures, we used aBSREL (adaptive branch-site random effects likelihood; [94]), a method to test for lineage-specific signals of positive selection within a gene family phylogeny.

With the concatenate of 98 single-copy ortholog protein sequences, we constructed an ML phylogeny with PhyML [95] using a JTT+G+I model for substitution, selected by SMS analysis [96] according to Akaike information criterion, and Bootstrap resampling for estimating branch support. Species divergence times were estimated using a Bayesian Markov chain Monte Carlo (MCMC) approach, calculating the approximate likelihood, as implemented in MCMCTree from the PAML 4.9 package [97], using the minimum-divergence ages reported for Hymenoptera, Coleoptera, Thysanoptera and Isoptera clades [98]. We ran two parallel MCMC chains for 11 million generations, sampling every 500 generations and specifying an initial burn-in of one million generations. We confirmed convergence between the two chains using tracer v1.6 [99]. The concatenated single-copy ortholog alignment, the species tree, and the dated phylogeny are available in TreeBase (submission ID 23203). 

Previous studies found independent events of acceleration in substitution rates associated with a convergent mutualistic lifestyle in ants of the *Pseudomyrmex* genus [37]. In order to test for any difference in the substitution rates between mutualists and generalists within our dataset, we analyzed the association between nutritional mutualism with fungi as a binary ecological trait, and evolutionary rates among the species of insects studied. To this end, we used TraitRareProp [100,101], which detects trait-dependent evolutionary rate shifts in sequence sites. Terminal branches were assigned to a state of obligate mutualism, 1 or 0. All the attine ants (*Cyphomyrmex costatus, Atta cephalotes, Acromyrmex echinatior, Trachymyrmex zeteki*), ambrosia beetles *(X. glabratus* and *E.* nr. *fornicatus*), and *Dendroctonus ponderosae* received a mutualist state assignment, based on the knowledge of their mutualistic symbiosis with fungi. Due to the intermediate nature of the mutualism in *D. ponderosae*, we ran this same analysis considering only strict fungus farming species and excluding this species, to analyze the differences between these two types of mutualism (Figure S5). Additionally, we conducted a TraitRateProp analysis with sociality as ecological binary trait, considering eusocial (*Apis mellifera*, *Polistes dominula*, *Cerapachys birori*, *Atta cephalotes*, *Acromyrmex echinatior*, *Trachymyrmex zeteki*, *Cyphomyrmex costatus*, *Zootermopsis nevadensis*, and *Cryptotermes secundus*) and facultative eusocial species (*X. glabratus* and *E.* nr. *fornicatus*) as social insects.

Finally, GO term enrichment analysis was performed using topGO R package (https://bioconductor.org/packages/release/bioc/html/topGO.html) to evaluate the possibility of over-represented terms among the genes with signals of positive selection, using all genes present at the transcriptome of each organism as the gene universe. To assess possible GO terms over-represented among the genes with decreased or increased evolutionary rates associated with mutualism, all the single-copy ortholog genes were used as the gene universe. In both cases we focused on the biological process (BP) category and used Fisher’s exact test for the statistics. A *p*-value of ≤ 0.01 was considered the threshold to select significant results. 

### 2.8. Differential Expression Analysis

We analyzed the differential expression patterns of beetle and fungal genes between the three datasets of *E.* nr. *fornicatus*–*Fusarium euwallaceae* complex: abdomen, head-thorax and whole body. We used RSEM 1.2.17 [102] to quantify the gene expression and test for significant expression differences between tissues. 

Paired unassembled reads of each sample were aligned to the unigene transcript sequences with Bowtie 2 [103]. From this mapping, we obtained a matrix of raw read counts and TMM-normalized FPKMs (i.e., trimmed mean of M values-normalized fragments per kb of transcript per million reads mapped) expression values per unigene transcript. For the differential expression analysis, we considered that each sample in our data represented a single biological replicate and used edgeR package [104] to calculate the negative binomial dispersion across conditions from the read counts of genes, using a dispersion parameter of 0.1. For the test of significant tissue enrichment, p values from the differential expression analyses were adjusted for false discovery rate (FDR) with the Benjamini and Hochberg correction [105]. Only genes with *p*-adjusted ≤ 0.01 were considered as differentially expressed genes. According to this and total expression profiles, each gene then received a category of organ localization: organ-specific, organ-enriched, or whole body. 

## 3. Results

We generated two *de novo* transcriptome assemblies of 221.3 and 110.6 Mb, for *E.* nr*. fornicatus* and *X. glabratus* respectively (Table 1). 82% of *E.* nr. *fornicatus* and 79% of *X. glabratus* predicted unigenes were functionally annotated with either InterProScan5 or BLASTp. The genome with the highest proportion of homologous genes was *D. ponderosae* for both ambrosia beetle transcriptomes. 

Based on the comparison of unassembled reads and assembled transcripts against the fungal databases, we identified 16,738 putatively fungal unigenes obtained from *X. glabratus* and 10,925 from *E.* nr. *fornicatus* transcriptomes, of which 95.03 and 90.94% showed homology with *Raffaelea lauricola* and *Fusarium euwallaceae* proteomes respectively and were considered as separate datasets for further analyses.

Enrichment analysis of fungal-related sequences against *F. euwallaceae* and *R. lauricola* proteomes revealed 10 GO categories enriched in both fungus-like gene sets within *E.* nr. *fornicatus* and *X. glabratus* transcriptomes, involved in constitutive functional categories such as translation, vesicle transport and exocytosis, splicing, ATP hydrolysis, proteolysis, and protein folding (See Table S5). Particularly, the *X. glabratus* fungal sequence set was enriched in the isoprenoid biosynthetic process category (GO:0008299).

### 3.1. Gene Family Analysis

A gene family analysis for *E.* nr. *fornicatus* and *X. glabratus* transcriptomes was conducted with genomes from 14 other insects (TableS4: A3), to evaluate the genomic signals associated with the evolution of mutualistic lifestyle and social behavior in ambrosia beetles. 

A total of 107,701 gene family clusters were retrieved, where 2475 families were shared by most of the analyzed species (~90%) and 10,565 were moderately conserved among taxa (~60%). The core of homologous genes conserved in all compared species consisted of 946 gene families.

*E.* nr. *fornicatus* and *X. glabratus* were the datasets with the largest proportion of unique gene families (23,412 and 24,945 respectively), mostly consisting of a single member that may be attributable to the presence of gene isoforms in both transcriptome datasets. Furthermore, both ambrosia beetle species shared 6946 gene families, of which 1714 are potentially unique for this clade and lack homologues in any other of the compared genomes, while containing mainly unclassified proteins, as well as genes related to the cellular process (GO:0009987) and cellular component organization or biogenesis (GO:0071840) functional categories. 

Moreover, we observed 2238 clusters shared by ambrosia beetles and eusocial insects. Within this group the most represented subcategories were signal transduction (GO:0007165) and transcription, DNA-dependent (GO:0006351). Two of the largest gene families of this eusocial insect and ambrosia beetle-shared group, consisting of 102 and 163 members, corresponded to nervous system-associated genes, such as the family of kainate receptor [106] and Down syndrome cell adhesion molecule-like protein (Dscam; [107]), respectively. 

### 3.2. Genes under Selection in the Xyleborini Ambrosia Beetle Lineage

We evaluated whether selective pressures (dN/dS) vary among the branches of each gene family phylogeny with aBSREL. We identified 1425 terminal branches with signals of positive selection (*p* ≤ 0.05 in Likelihood ratio tests for episodic positive selection, Holm-Bonferroni corrected) in gene family phylogenies across 16 insect species (Figure S1). From these, we found 176 and 166 coding genes under positive selection in *X. glabratus* and *E.* nr. *fornicatus* respectively (Tables S6 and S7, Figures S2–S4). 

Among all the genes under selection, there were 80 Interpro accessions shared by positively selected genes of both species. These genes were mainly involved in sugar transport (facilitated trehalose transporter Tret1; [108]), abiotic stress response (Heat shock protein 70 family; [109]), transcriptional regulation (protein drumstick, E3 ubiquitin-protein ligase Parkin and ZZ-type zinc finger-containing protein 3; [110,111,112]), G-protein receptors (GPCRs), signal transduction (Rab3 GTPase-activating protein, Synembrin-A, tyrosine kinase receptor Cad96Ca, tuberin and G-protein coupled receptor 143; [113,114,115,116,117]), development and muscular morphogenesis (Afadin, Ecdysteroid kinase, ankyrin repeat domain-containing protein 16-like, unconventional myosin-XVIIIa, Hemicentin-1; [118,119,120,121]), and several genes involved in the development and function of the nervous system (Nesprin-1, SLIT-NTRK protein 1, neuroligin-2, neuronal PAS-domain-containing protein 4, Amphysin; [122,123,124,125]). 

Among the genes under selection in the *E.* nr. *fornicatus* transcriptome, 100 were expressed in the whole body, 45 were enriched or specific for abdomen and 19 for head-thorax (see Table S6 for more details). Signals of selection were found on the sex peptide receptor [126,127] and galactosylgalactosylxylosyl protein 3-beta-glucuronosyltransferase P (GlcAT-P; [128]) genes, which are exclusively transcribed on the head-thorax. Particularly, genes under selection are significantly enriched (*p* ≤ 0.01 in Fisher’s exact test) in the GO functional categories of fructose metabolism (GO:0006000) in *E.* nr. *fornicatus* and apoptotic DNA fragmentation and protein processing (GO:0016485, GO:0006309) in *X. glabratus* transcriptomes, respectively. 

### 3.3. Changes in Evolutionary Rates Related to Fungus-Farming Mutualism and Sociality

**Figure 1:**
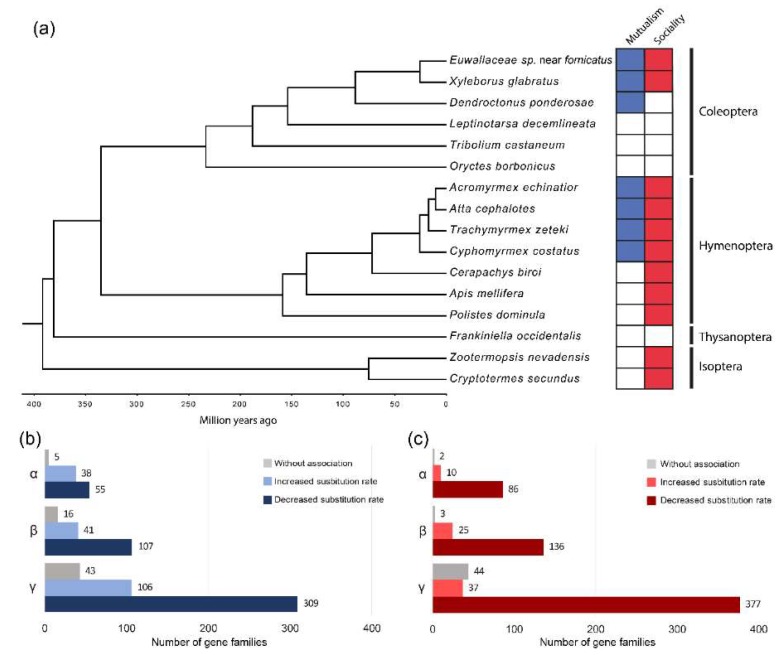
(**a**) Time-calibrated phylogeny based on 98 sequences from single-copy orthologs and mutualist-social behavior categories. (**b**) Single copy ortholog groups with substitution rate variations associated with mutualistic nutritional symbiosis with fungi. (**c**) Single copy ortholog groups with substitution rate variations associated with social behavior. α: Isoptera + Thysanoptera + Hymenoptera + Coleoptera; β: Thysanoptera +Hymenoptera + Coleoptera; γ: Coleoptera.

The species tree obtained from the phylogenetic analysis maintains all insect order clades as monophyletic groups, with bootstrap support values from 0.95 to 1 in all branches. The estimated age of divergence between Xyleborini species is approximately 25.11 ± 13.31 million years ago (mya), and 88.56 ± 30.36 mya since divergence from *D. ponderosae* (Figure 1A). In the context of the species phylogeny, we evaluated the potential association of mutualism and sociality with variation in molecular substitution rates between species. The TraitRateProp analysis was performed in three groups of single-copy orthologs: considering all species (α, 98 orthologs), excluding species from the Isoptera order (β, 164 orthologs), and only considering the Coleoptera order (γ, 468 orthologs). The analysis of the latter subset of orthologs constitutes the direct comparison between the two ambrosia species (as mutualists and facultative eusocial species) and other beetles.

The greatest proportion of single-copy orthologs in all the groups revealed a significantly negative association (*p* ≤ 0.05 in chi-squared likelihood ratio test) between the rate of sequence evolution and the mutualistic lifestyle, as well as the social category (Tables S8 and S9). Although this association was predominantly negative (genes with a slower molecular rate of evolution in mutualistic and social species), genes with a positive association were also found, showing an accelerated molecular evolutionary rate in mutualistic and social lineages (Figure 1B,C). No particular GO functional category was observed to be significantly enriched in single-copy orthologs with either an increased or decreased rate associated with the two traits.

All genes representing a negative association between mutualism and evolutionary rate likewise exhibited a negative association with sociality. Furthermore, there was a larger number of gene families positively related to mutualism than to sociality. As trait-dependent accelerations in the rate of molecular evolution can reflect events of either positive or relaxed selection, we analyzed the evidence of natural selection in proteins with a positive association between a trait and substitution rate. We thus identified several genes with evidence of positive selection and convergent evolutionary rate acceleration associated with sociality, mutualism, and both traits (Table 2).

## 4. Discussion

Mutualistic symbiotic interactions represent a source of evolutionary novelty, and consequently modify the genomes of the species involved [129,130,131]. Our study provides novel insights on the molecular and genomic processes involved in the evolution of sociality and obligate fungus farming in ambrosia beetles of the Xyleborini tribe. Based on natural selection and substitution rate analyses of ortholog gene families, several genes and pathways potentially associated with these evolutionary transitions were identified.

### 4.1. Evolutionary Changes Possibly Associated with Nutritional Symbiosis with Fungi

The evolution of fungus farming in ambrosia beetles is associated with a dietary specialization related to the low content of relevant nutrients in the phloem-based diet of their bark beetle ancestors. Such specialization implies the increase of dietary nitrogen [9,132] and the acquisition of other important molecules such as sterols, required for metamorphosis and reproduction ([9,10,133]).

Among the genes with signals of positive selection in the transcriptomes of *E.* nr. *fornicatus* and *X. glabratus,* fructose metabolism and proteolysis functional categories were found to be enriched. Nitrogen can be acquired from a protein-rich diet through proteolysis, therefore selection in genes encoding proteolytic enzymes, such as trypsins, could constitute an evolutionary adjustment to the changed nitrogen availability in a fungus-based diet. Some adjustments related to nutrient acquisition have been observed in fungus-farming ant genomes. For instance, the gene losses in arginine biosynthetic pathways or the reduction in the serine protease gene family have been observed in ants, and are thought to be associated with variations in dietary nitrogen following fungus farming evolution [68,71].

On the other hand, signals of positive selection were observed in genes related to carbohydrate metabolism and transport in both ambrosia beetle species. First, 6-phosphofructo-2-kinase/fructose-2,6-bisphosphatase (PFKFB) is an enzyme that catalyzes the synthesis and degradation of fructose 2,6-bisphosphate, which functions as a signal molecule for glycolysis and gluconeogenesis modulation [134]. Secondly, trehalose transporter Tret1, which was also found to be under positive selection, regulates levels of trehalose (as the main storage sugar) in insect hemolymph, as well as its release from the body fat and incorporation of trehalose as a carbon source into other tissues such as muscle and testis [108]. Trehalose also appears to have a role for buffering sugar fluctuation in response to food composition and quantity [135], both of which could potentially change in a transition from a phloem-based diet to a fungal-based diet.

Molecular evolution associated with changes in diet as a consequence of farming and domestication has been widely studied in humans (e.g., [136,137,138,139]). In humans, these mechanisms involve the evolution of genes related to metabolism, nutrient homeostasis, digestion, sensory perception, appetite control, and morphological development of the digestive system [139]. In addition, many changes related to nutrient acquisition in the genomes of fungus-farming ants [34,68,71], as well as changes in mass-specific metabolic rates through fungus-farming evolution stages in Attini ants, have also been documented [21]. Therefore, selection on genes that participate in energy and nutrient homeostasis may be associated with dietary specialization after fungi farming evolved in the Xyleborini tribe.

### 4.2. Evolutionary Changes Possibly Related to the Evolution of Behavior and Sociality.

Many behavioral adaptations can be recognized in *X. glabratus* and *E.* nr. *fornicatus* since the divergence and radiation of the Xyleborini tribe. These beetles bore deep into the wood instead of bark [8], inbreed [4], and farm fungi [3,4], involving activities such as pathogen monitoring and control and selective use of substrate for fungi farming [10,22]. There is also evidence that suggests that these ambrosia beetles could display a facultative eusocial system [11], characterized by overlapping generations, cooperative care of the offspring, and age polyethism [12,13,14].

As previously mentioned, the general hypotheses about evolution of sociality suggest that the co-option of conserved gene networks, as well as selection, and fast protein evolution are involved in the emergence of social behaviors and phenotypes [21,22,23], while natural selection through inclusive fitness should play a critical role in the evolution of sociality [31,32]. We found signals of positive selection in several genes involved in transcriptional regulation, reproduction, signal transduction development, and nervous system functioning, which could be associated with the transition to sociality in ambrosia beetles, as well as with other major changes in behavior since the divergence of this lineage.

The first group of genes under selective pressure found to be associated with social behavior were related to the transcriptional regulation of gene expression. Previous studies in eusocial organisms such as bees suggest that evolutionary changes in gene regulation are critical for the appearance of specialized phenotypes [140,141] and the production of social traits such as social foraging, reproductive dominance, and alloparental care [24,32,142], the latter having been observed in ambrosia beetles [8]. Genes involved in transcriptional regulation and with signals of positive selection in *X. glabratus* and *E.* nr. *fornicatus* identified in this work are related to digestive tract development (protein drumstick), mitochondrial integrity and oxidative stress response (E3 ubiquitin-protein ligase parkin), chromatin organization (ZZ-type zinc finger-containing protein 3), and development of olfactory and sensory organs (basic helix-loop-helix *amos* transcription factor and suppressor of hairless protein). Suppressor of hairless gene also shows an accelerated evolution in both ambrosia beetles compared to other species of the Coleoptera order, suggesting that this gene has an important role in the adaptation of these species. Since olfactory and sensory systems regulate many insect behaviors [143,144], such as the communication and recognition between individuals in social organisms [31], adaptive signals observed in these genes could be involved in the evolution of behavior and sociality within this group. Moreover, transcriptional regulation can be related to the evolution of other apomorphic traits in these species, therefore the transcriptional profiles need to be analyzed across different members of an ambrosia beetle colony to test these hypotheses.

The second group relates to genes involved in reproductive behavior. Sex peptide receptor was observed to be under positive selection in *E.* nr. *fornicatus*. This gene plays a role in female post-mating behavior shifts [126,127] and the release of stored sperm for fertilization [145] in *D. melanogaster.* Gene networks regulating reproduction, and particularly those regulating the changes between reproductive and non-reproductive behavioral stages, are predicted to have pleiotropic effects on reproductive traits and social behaviors such as age polyethism and parental care [23,146]. The changes in this gene could be related to delayed reproduction and dispersal, promoting altruistic behaviors as observed in the facultative eusocial beetle *Xyleborinus saxesenii* [13].

The third group comprises genes implicated in GPCRs signal transduction. Ligands of GPCRs include hormones, peptide and non-peptide neurotransmitters, odorant molecules, growth factors, and light, and signaling through these proteins is important for controlling processes such as development, reproduction, behavior, and feeding [147]. Chemical communication is widely recognized to have a critical role in social behavior in hymenopterans [148] and termites [33]. Concerning ambrosia beetles, recent findings document the production of and response to two volatiles compounds (2-heneicosanone and 2-tricosanone) by three cryptic species of *E.* near *fornicatus* [polyphagous shot hole borer (PSHB), Kuroshio shot hole borer (KSHB), and tea shot hole borer (TSHB)], [149]. These compounds are produced in unique ratios and work like pheromones, showing species-specific attraction and repellency. Previous works have also detected these two volatile compounds in the mandibular gland secretions of the stingless bee *Scaptotrigona postica* [150], and in the subterranean termite *Reticulitermes flavipes* [151], two insect species which display high levels of sociality. Therefore, molecular adaptations associated with communication between members of a population could be playing an important role in the coordination of several behaviors, and in the emergence of sociality in ambrosia beetles [31].

The fourth group includes development-associated genes, such as the cornichon protein. It has been proposed that the evolution of developmental pathways is important for increasing termite eusociality, which is characterized by differentiated castes represented by distinct developmental stages [33]. Even though ambrosia beetles do not display caste differentiation, they present a division of labor between larvae and adults, which in turn could be related to selective pressures towards future cast differentiation. Alternatively, developmental adaptation could play an important role in the formation of specialized structures, such as mycangia, as well as other morphological changes present in these species, like the increase of sexual dimorphism and the decrease in size, since diverging from its common ancestor with *D. ponderosae*.

The last group of genes associated with sociality and under selective pressure was involved in the nervous system and sensory functions. The signals of positive selection on genes involved in the development and function of the nervous system in both *E.* nr. *fornicatus* and *X. glabratus* suggest an adaptive evolution in the ambrosia beetles towards changes in behavior. Among these genes, we found several proteins conserved in mammals and insects required for synaptogenesis (amphysin, liprin, neuroligin-2, neurexin-4, SLIT-NTRK protein 1, GlcAT-P; [128,152,153,154], nervous system development (neuronal PAS-domain-containing protein 4, Neural/ectodermal development factor, IMP-L2; [155,156], neurotransmitter transport, (sodium neurotransmitter symporter; [157] and, as previously mentioned, development of sensory organs (basic helix-loop-helix *amos* transcription factor and suppressor of hairless protein [158,159,160]. Neuroligin-2 has been directly associated with social behavior in *D. melanogaster* [161] and GlcAT-P shows caste-specific transcriptional patterns during brain development in *Apis mellifera* [128]. We found that GlcAT-P is expressed in the head-thorax of *E.* nr. *fornicatus*, which points to an adaptation related to social behavior in ambrosia beetles.

Furthermore, two of the largest gene families shared by ambrosia beetles and eusocial insects are involved in the function of the nervous system (a receptor that controls synaptic transduction: kainate receptor [106], and the neuronal guidance factor Down syndrome cell adhesion molecule-like protein (Dscam; [107]) observed to have effects on locomotor behavior and fecundity respectively, further suggesting the importance of genes associated with behavior evolution in ambrosia beetles.

### 4.3. Possible Causes of Accelerated or Decreased Evolutionary Rates

We found significant differences in the substitution rates between mutualistic and non- mutualistic, as well as social and nonsocial, insects. Most of the single-copy orthologs showed a slower evolutionary rate in social and mutualist phylogenetically independent lineages. More genes showed slower evolutionary rates in social and strict fungus-farming lineages than in general mutualistic ones (Figure 1 and Figure S5). Because all strict fungus farming considered display social behaviors, the genes that show this trait decrease in their evolutionary rates are more likely to be associated to the evolution of sociality than with mutualism.

Slowly evolving proteins could be under strong purifying selection during the evolution of social lineages, reflecting a constrained protein evolution in these species. Low evolutionary rates associated with highly specialized sociality have been previously observed in bees with different levels of social organization [140], and in honey bees compared to other insects such as fruit flies [56]. This is in general agreement with the ‘genetic toolkit’ hypothesis, suggesting a set of highly conserved genes but an increased gene regulation during social evolution.

On the other hand, we observed several single-copy orthologs with accelerated rates related to mutualism and strict fungus framing. Accelerated rates could be a result of a positive or relaxed selection, but also a consequence of nonadaptive evolution, such as mechanisms related to demography due to genetic drift. However, the changes observed in global dN/dS ratios between mutualistic and non-mutualistic lineages (Figure S6) strongly suggest that the evolutionary rate acceleration related to mutualism is mainly due to changes in selective pressures acting on these genes. Some of these single-copy ortholog genes provide evidence of positive selection (Table 2), pointing to a convergent adaptive evolution towards this lifestyle, as well as the evolution of social organization.

### 4.4. Transcriptional Fungal Profiles

Different fungal species establish symbiotic interactions with bark and ambrosia beetles, having several levels of promiscuity and specificity [162,163]. In natural conditions, beetles are exposed to several microbial species and present multipartite interactions with different fungi. It has been proposed that specificity in these interactions largely depends on environmental factors such as temperature [164,165], as well as on differences in dietary benefits for beetles [9,162] in different life-cycle and gallery formation stages [166]. Therefore, exploring the functional capabilities of the different symbionts is needed for evaluation of these hypotheses.

Functional profiles of fungus-like genes transcribed in the body of *E.* nr. *fornicatus* and *X. glabratus* share most of the enriched functional categories. These categories can be related to fungus growth, but also to their interaction with the beetles. Particularly, the Isoprenoid biosynthesis GO category was present in fungus-like transcriptome from both types of ambrosia beetles, but enriched only in the body of *X. glabratus.* This biosynthetic process is needed for the synthesis of sterol, as well as other terpenoids. Besides, many of the scolytid aggregation pheromones are isoprenoids [167,168], including ipsdienol, ipsenol, and frontalin [169]. In particular, (1S,4R)- p-menth-2-en-1-ol (also known as quercivorol) is documented to function as an attractant kairomone for members of the *E.* nr. *fornicatus* species complex, and this compound is likely produced by the symbiotic fungi [149]. This aggregation compound has been identified in other ambrosia beetle species like *Ewuallacea* spp. and *Platypus quercivorus* (Coleoptera: Platypodidae) [170]. Therefore, the enrichment of this category reflects that these molecules could have an important role in establishing fungus–beetle interaction.

## 5. Concluding Remarks

Evolutionary transition to mutualism and social organization in ambrosia beetles appears to involve protein changes driven by natural selection that could constitute diet-related and social behavior adaptations to the new selective pressures of fungus-farming lifestyles. Selection appears to target different components with similar pathways to those that have been reported in different insect lineages for the transition to mutualism/sociality.

Moreover, across-lineage comparison allowed us to identify the convergent changes in protein evolution related to sociality and mutualism transitions. We observed conserved genes with a decreased molecular evolution, mainly associated with a sociality trait, and an accelerated evolution related to selective pressures across the mutualistic lineages studied.

Comparative analyses of transcriptomic and genomic data prevented us from performing a more detailed search of evolutionary signals, such as expansion and contraction of gene families or synteny. Future genomic analysis within the Scolytinae subfamily and Xyleborini tribe is needed to describe the evolution of mutualism, and to test the social evolution hypotheses, considering the differences between independent origins of these traits. Additionally, studies on the transcriptomics of beetles with comparable replicates between species, development stages, and sexes are fundamental to assess the detailed role of transcriptional gene regulation in the mechanisms underlying these complex traits.

## Figures and Tables

**Table 1:** Assembly and annotation statistics for the two ambrosia beetle transcriptomes sequenced in this study.

	*Euwallacea* sp. near *fornicatus*	*Xyleborus glabratus*
Total high-quality paired reads	61,919,467	68,243,721
Total number of assembled transcripts	248,739	150,163
N50 (bp)	1636	1261
Total assembled bases	221,342,598	110,621,067
Average contig length (bp)	889.86	736.67
Total number of predicted unigenes	68,490	46,814
InterproScan5 annotated unigenes	56,315	34,612
BLASTp annotated unigenes	55,115	37,188

**Table 2:** Genes with trait-dependent accelerations in the rate of molecular evolution, and with evidence of positive selection.

Cluster ID	Association with	BLASTp Annotation	Ortholog Group
199572_KRT80184	Mutualism	methylosome subunit pICln	α
199849_KRT80461	Mutualism	Rab-protein 6 (Rab6)	α
200376_KRT80988	Mutualism	ribosomal RNA small subunit methyltransferase NEP1	α
203911_KRT84523	Mutualism	phosphatidylethanolamine-binding protein homolog F40A3.3	α
198969_KRT79581	Both	ribosome biogenesis regulatory protein homolog	α
201287_KRT81899	Sociality	ubiquitin carboxyl-terminal hydrolase 30	α
203022_KRT83634	Sociality	phosphoacetylglucosamine mutase-like	α
180851_KRT82102	Both	NADH dehydrogenase (ubiquinone) 1-alpha subcomplex 9, 39kDa (Ndufa9)	β
181368_KRT82619	Both	Bax inhibitor 1 (BaxI1)	β
182416_KRT83667	Both	vesicle transport through interaction with t-SNAREs homolog 1A	β
182600_KRT83851	Both	UDP-N-acetylglucosamine-dolichyl-phosphate N-acetylglucosaminephosphotransferase	β
183558_KRT84809	Sociality	ribosome-recycling factor, mitochondrial	β
185607_KRT86858	Sociality	malate dehydrogenase, mitochondrial	β
176873_KRT78124	Mutualism	poly (ADP-ribose) glycohydrolase ARH3	β
177777_KRT79028	Mutualism	DNA replication complex GINS protein SLD5	β
181622_KRT82873	Mutualism	ubiquitin domain-containing protein 1/2	β
185046_KRT86297	Mutualism	MFS-type transporter C6orf192 homolog	β
100143_KRT79886	Both	cysteine-rich with EGF-like domain protein 2	γ
101937_KRT81680	Both	Cornichon protein	γ
103130_KRT82873	Both	ubiquitin domain-containing protein 1	γ
99990_KRT79733	Both	suppressor of hairless protein	γ
100907_KRT80650	Both	transmembrane protein 127	γ
99195_KRT78938	Both	voltage-dependent anion-selective channel protein 2	γ

## Supplementary Materials

The following are available online at http://www.mdpi.com/2075-1729/9/1/2/s1, Table S1: Overview of Illumina short read sequencing for the two ambrosia complexes and quality control, Table S2: External genomic fungal data sources used for the first fungus-like sequences screening, Table S3: Assembly and annotation statistics of the ambrosial complexes transcriptomes generated in this study, Table S4: External genomic insect data sources, Table S5: GO term counts of fungus-ike sequences retrieved from *E*. nr. *fornucatus* and *X. glabratus*, Table S6: Summary of genes in *Euwallacea* sp. near *fornicatus* under positive selection, Table S7: Summary of genes in *Xyleborus glabratus* under positive selection, Table S8: Summary of conserved gene families with evolutionary rate associated to mutualism, Table S9: Summary of conserved gene families with evolutionary rate associated to sociality, Figure S1: Genes under selection identified with aBSREL analysis (*p* ≤ 0.05 in Likelihood ratio test for episodic diversifying positive selection at Holm-Bonferroni corrected) for each insect species, Figure S2: Gene Ontology (GO) Biological Process categories associated with the genes with signals of positive selection in *X. glabratus* and *E*. nr. *fornicuatus*, Figure S3: Gene Ontology (GO) Cellular Component categories associated with the genes with signals of positive selection in *X. glabratus* and *E*. nr. *fornicuatus*, Figure S4: Gene Ontology (GO) Molecular Process categories associated with the genes with signals of positive selection in *X. glabratus* and *E*. nr. *fornicuatus*, Figure S5: Comparison between TraitRateProp analysis. Single copy ortholog groups with changes in its substitution rate associated with strict fungus farming (Attini and ambrosia beetles species) and with general nutritional mutualism with fungi (Attini ants, ambrosia beetles and *D. ponderosae* species).
