# Supplementary material for: Genomic Signals of Adaptation towards Mutualism and Sociality in Two Ambrosia Beetle Complexes
Source: Life (Basel). 2018 Dec 22;9(1):2. doi: 10.3390/life9010002 (PMC6463014; doi:10.3390/life9010002)
Supplement: Supplementary file 1 [file life-09-00002-s001.zip › Supplementary Material/Supplementary Material 01 (Tables S1-S4, and Figures S1-S6).docx]

**Table S1.** Overview of Illumina short read sequencing for the two ambrosia complexes and quality control.

| **Description** | **SRA Accession** | **Paired-end sequences** | **HQ* Reads** | **Total of paired raw reads** | **Total HQ reads** |
| --- | --- | --- | --- | --- | --- |
| *X. glabratus* (complete body) | EI1XG1SS01 | 19,294,049 | 14,266,778 | 83,042,239 | 68,243,721 |
|  | EI1XG1SS02 | 22,972,535 | 17,004,930 |  |  |
|  | EI1XG1SS01 | 24,597,229 | 22,424,976 |  |  |
|  | EI1XG1SS02 | 16,178,426 | 14,547,037 |  |  |
| *Euwallacea* sp. near *fornicatus* (abdomen) | EI1EF1SS01 | 78,509,427 | 70,554,673 | 182,822,888 | 161,919,467 |
| *E.* nr. *fornicatus* (head and thorax) | EI1EF1SS02 | 40,869,695 | 36,335,152 |  |  |
| *E.* nr. *fornicatus* (complete body) | EI1EF1SS03 | 63,443,766 | 55,029,642 |  |  |

* HQ: High quality

**Table S2.** External genomic fungal data sources used for the first fungus-like sequences screening

| **Species** | **Source** | **Acession** | **Data type** | **Reference** |
| --- | --- | --- | --- | --- |
| *Fusarium euwallacea* | NCBI | NHTE00000000.2 | genomic CDSs | [48] |
| *Fusarium temperatum* | NCBI | GCA_001513835.1 | genomic CDSs | [50] |
| *Ceratocystis eucalypticola* | NCBI | GCA_001513815.1 | genomic CDSs | [50] |
| *Ceratocystis platani* | NCBI | GCA_000978885.1 | genomic CDSs | https://www.ncbi.nlm.nih.gov/nuccore/814603118 |
| *Thielaviopsis musarum* | NCBI | GCA_001513885.1 | genomic CDSs | [50] |
| *Thielaviopsis punctulata* | NCBI | GCA_000968615.1 | genomic CDSs | [51] |
| *Acremonium alcalophilum* | JGI | 1184963 | genomic CDSs | https://genome.jgi.doe.gov/Acral2/Acral2.info.html |
| *Microascus trigonosporus* | JGI | 1015502 | genomic CDS | https://genome.jgi.doe.gov/Mictr1/Mictr1.home.html |
| *Ambrosiella xylebori* | SRA | SRR5865576 | Transcriptome | [49] |
| *Candida sp.* | NCBI | GCA_001950555.1 | genomic CDs | [52] |
| *Ceratocystiopsis brevicomi* | SRA | SRR5865566 | Transcriptome | [49] |
| *Fragosphaeria purpea* | SRA | SRR5865573 | Transcriptome | [49] |
| *Grosmannia penicillata* | SRA | SRR5865574 | Transcriptome | [49] |
| *Meyerozyma caribbica* | NCBI | GCA_000755205.1 | genomic CDSs | [53] |
| *Raffaelea albimanens* | SRA | SRR5865580 | Transcriptome | [49] |
| *Raffaelea ambrosiae* | SRA | SRR5865577 | Transcriptome | [49] |
| *Raffaelea arxii* | SRA | SRR5865578 | Transcriptome | [49] |
| *Raffaelea lauricola* | SRA | [SRR5865575](https://trace.ncbi.nlm.nih.gov/Traces/sra/?run=SRR5865575) | Transcriptome | [49] |
| *Raffaelea sp* | SRA | SRR5865582 | Transcriptome | [49] |
| *Raffaelea sulphurea* | SRA | SRR5865581 | Transcriptome | [49] |
| *Saccharomycopsis fibuligera* | SRA | SRR3598378 | Transcriptome | https://www.ncbi.nlm.nih.gov/sra/?term=SRR3598378 |
| *Saccharomycopsis malangia* | SRA | DRR032483 | Transcriptome | https://www.ncbi.nlm.nih.gov/sra/?term=DRR032483 |
|  |  |  |  |  |

**Table S3.** Assembly and annotation statistics of the ambrosial complexes transcriptomes generated in this study.

|  | **Number of**  **Transcripts** | **N50** | **Total assembled bases** | **Average contig length** | **Filtered transcript unigenes*** | **InterproScan5 annotated unigenes** |
| --- | --- | --- | --- | --- | --- | --- |
| *Xyleborus glabratus* | 150,163 | 1,261 | 110,621,067 | 736.67 | 46,814 | 34,612 |
| Fungal sequences from *X. glabratus* | 921 | 520 | 402,577 | 437.11 | 16,738 | 15496 |
| *Euwallacea* sp. | 248,739 | 1,636 | 221,342,598 | 889.86 | 68,607 | 56,315 |
| Fungal sequences from *Euwallacea* sp. | 9959 | 477 | 4,308,891 | 432.66 | 10,925 | 7628 |

***** Filtered transcript unigenes through minimum length of translated product, and second fungus-like sequences screening as are described in methods.

**Table S4.** External genomic insect data sources.

| **Species** | **Order** | **Source** | **Accession** | **A1** | **A2** | **A3** | **Ref.** |
| --- | --- | --- | --- | --- | --- | --- | --- |
| *Aethina tumida* | Coleoptera | NCBI | GCF_001937115.1 | X | X |  | [60] |
| *Oryctes borbonicus* | Coleoptera | NCBI | GCA_001443705.1 | X | X | X | [61] |
| *Ignelater luminosus* | Coleoptera | [fireflybase.org](http://fireflybase.org) | Ignelater luminosus | X |  |  | [62] |
| *Anoplophora glabripennis* | Coleoptera | NCBI | GCF_000390285.2 | X | X |  | [63] |
| *Nicrophorus vespilloides* | Coleoptera | NCBI | GCA_001412225.1 | X | X |  | [60] |
| *Leptinotarsa decemlineata* | Coleoptera | NCBI | GCF_000500325.1 | X | X | X | [64] |
| *Photinus pyralis* | Coleoptera | [fireflybase.org](http://fireflybase.org) | Photinus pyralis | X |  |  | [62] |
| *Aquatica lateralis* | Coleoptera | [fireflybase.org](http://fireflybase.org) | Aquatica lateralis | X |  |  | [62] |
| *Dendroctonus ponderosae* | Coleoptera | NCBI | GCF_000355655.1 | X | X | X | [65] |
| *Tribolium castaneum* | Coleoptera | NCBI | GCF_000002335.3 | X | X | X | [66] |
| *Apis mellifera* | Hymenoptera | NCBI | GCF_000002195.4 | X | X | X | [67] |
| *Atta cephalotes* | Hymenoptera | NCBI | GCF_000143395.1 |  | X | X | [68] |
| *Cerapachys birori* | Hymenoptera | NCBI | GCF_000611835.1 |  |  | X | [69] |
| *Polistes dominula* | Hymenoptera | NCBI | GCF_001465965.1 |  |  | X | [70] |
| *Acromyrmex echinatior* | Hymenoptera | NCBI | GCF_000204515.1 |  | X | X | [71] |
| *Trachymyrmex zeteki* | Hymenoptera | NCBI | GCF_001594055.1 |  |  | X | [34] |
| *Cyphomyrmex costatus* | Hymenoptera | NCBI | GCF_001594065.1 |  |  | X | [34] |
| *Zootermopsis nevadensis* | Isoptera | NCBI | GCF_000696155.1 |  | X | X | [72] |
| *Cryptotermes secundus* | Isoptera | NCBI | GCF_002891405.1 |  |  | X | [33] |
| *Frankliniella occidentalis* | Thysanoptera | NCBI | GCA_000697945.2 |  |  | X | [60] |
| *Drosophila melanogaster* | Diptera | NCBI | GCF_000001215.4 | X | X |  | [73] |

A1: Used for AlignWise prediction; A2: Used for BLASTp analysis; A3: Included in evolutionary analysis

**Figure S1.** Genes under selection identified with aBSREL analysis (p ≤ 0.05 in Likelihood ratio test for episodic diversifying positive selection at Holm-Bonferroni corrected) for each insect species.


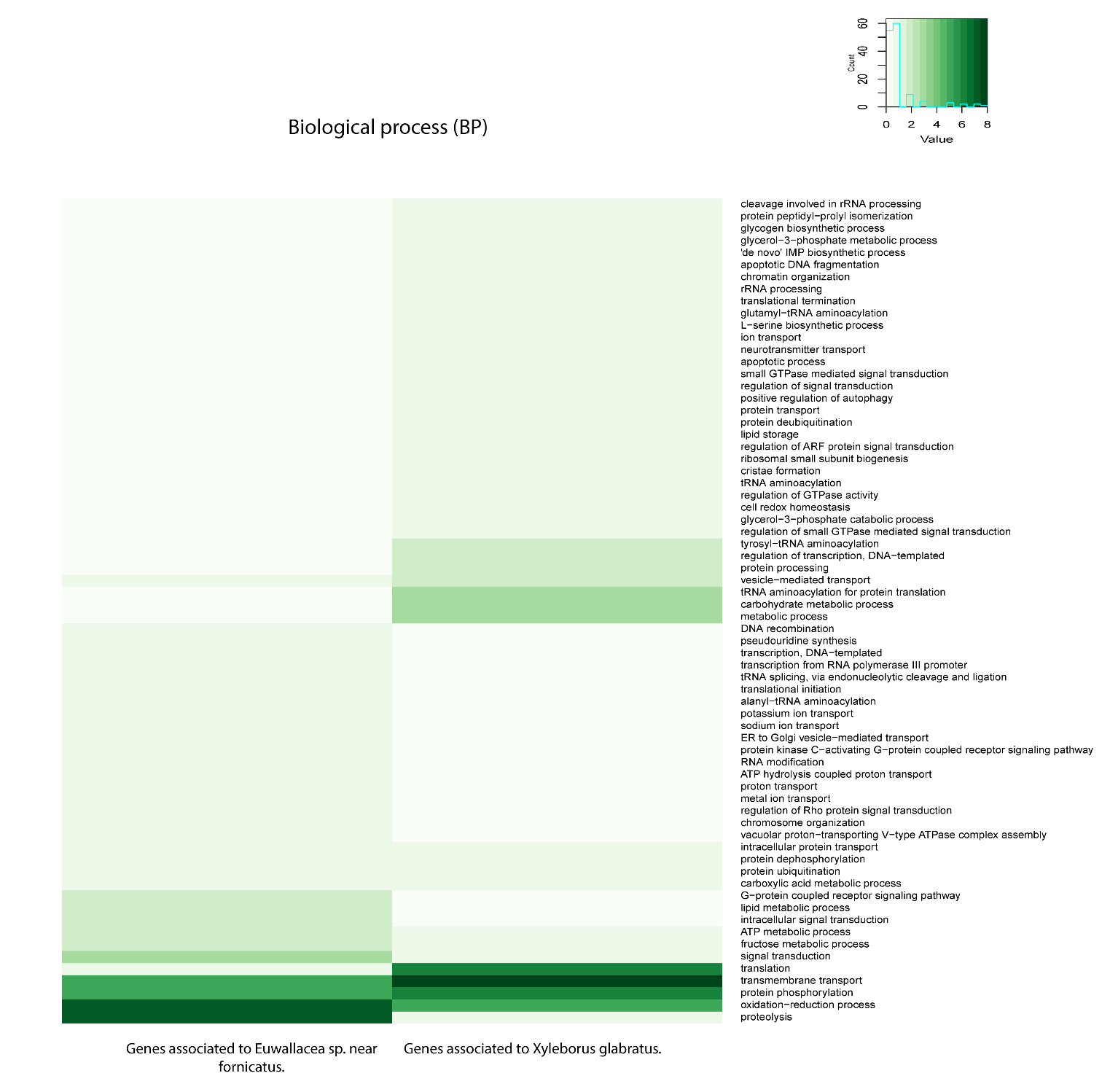


**Figure S2.** Gene Ontology (GO) Biological Process categories associated with the genes with signals of positive selection in *X. glabratus* and *E.* nr*. fornicuatus.* Light colors indicate less abundance and dark color high abundance genes under each category.


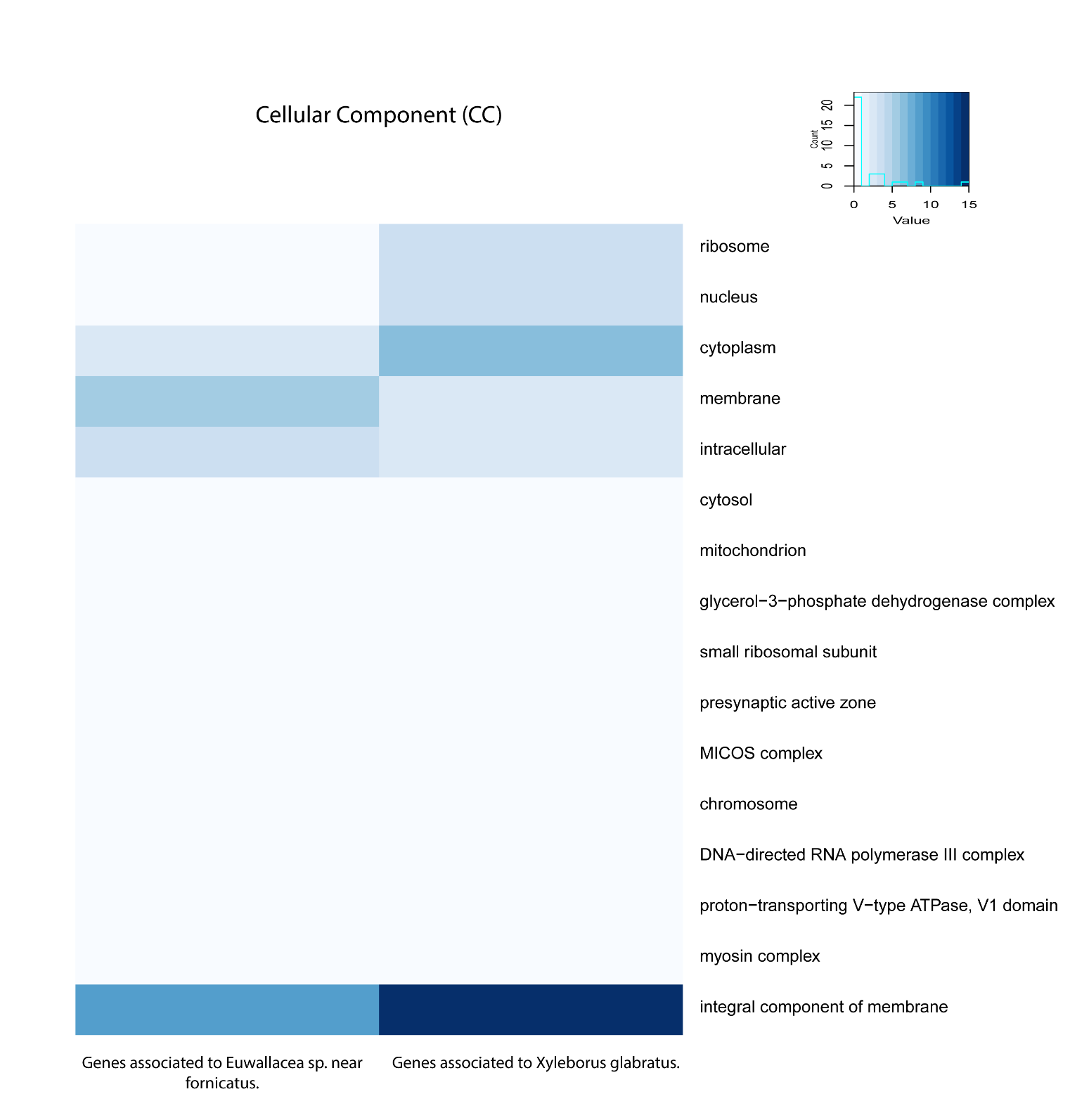


**Figure S3.** Gene Ontology (GO) Cellular Component categories associated with the genes with signals of positive selection in *X. glabratus* and *E.* nr. *fornicuatus.* Light colors indicate less abundance and dark color high abundance genes under each category.


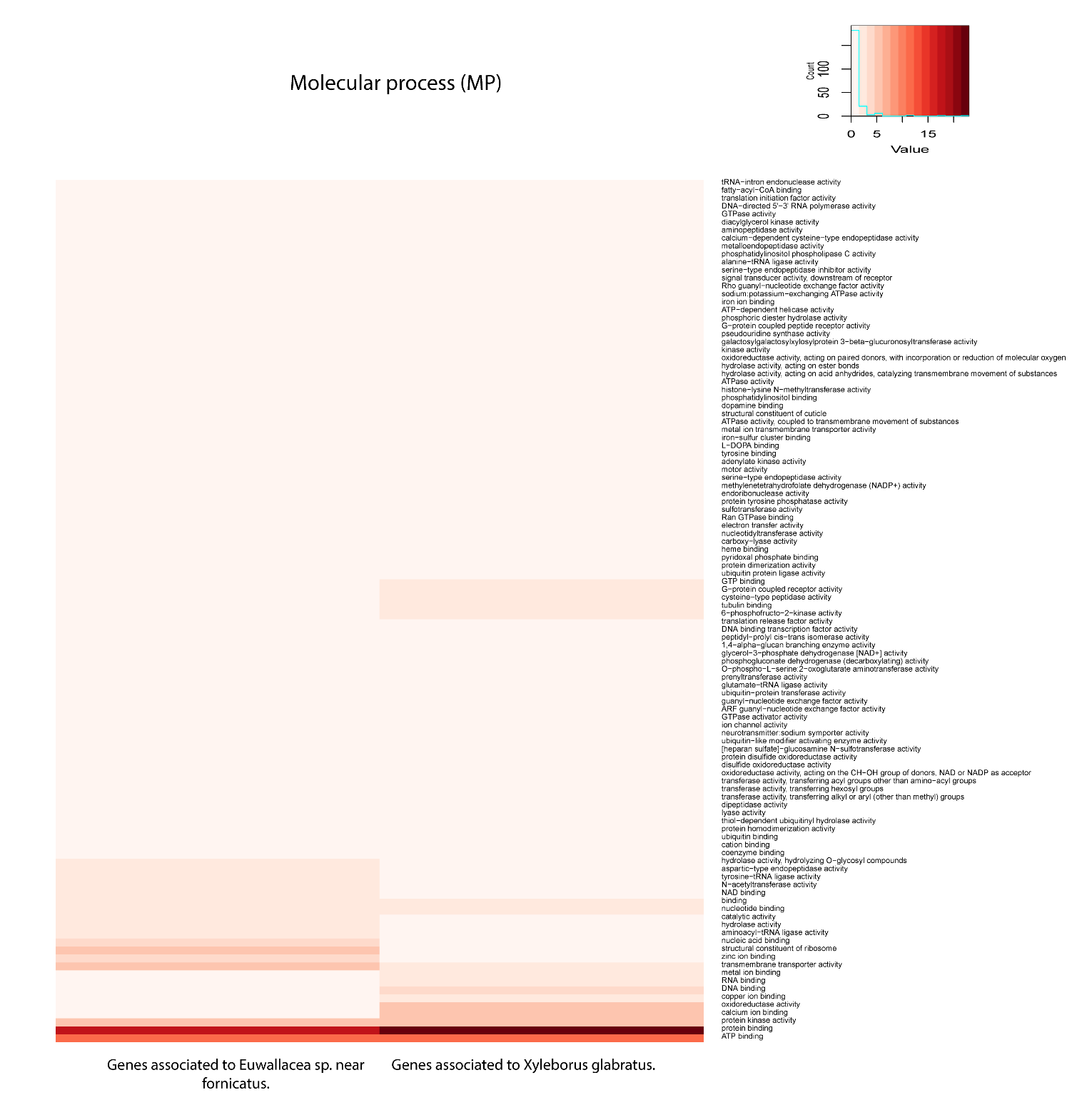


**Figure S4.** Gene Ontology (GO) Molecular Process categories associated with the genes with signals of positive selection in *X. glabratus* and *E.* nr. *fornicuatus.* Light colors indicate less abundance and dark color high abundance genes under each category.


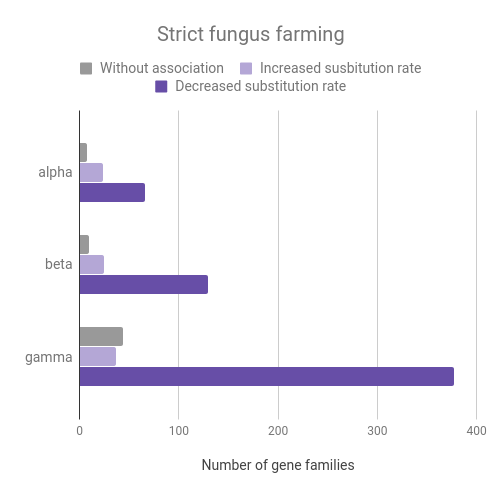

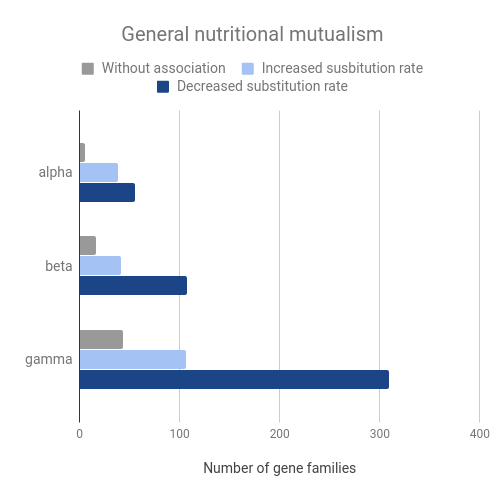


**Figure S5.** Comparison between TraitRateProp analysis. Single copy ortholog groups with changes in its substitution rate associated with strict fungus farming (Attini and ambrosia beetles species) and with general nutritional mutualism with fungi (Attini ants, ambrosia beetles and *D. ponderosae* species). α (alpha): Isoptera + Thysanoptera + Hymenoptera + Coleoptera; β (beta): Thysanoptera + Hymenoptera + Coleoptera; γ (gamma): Coleoptera.

**
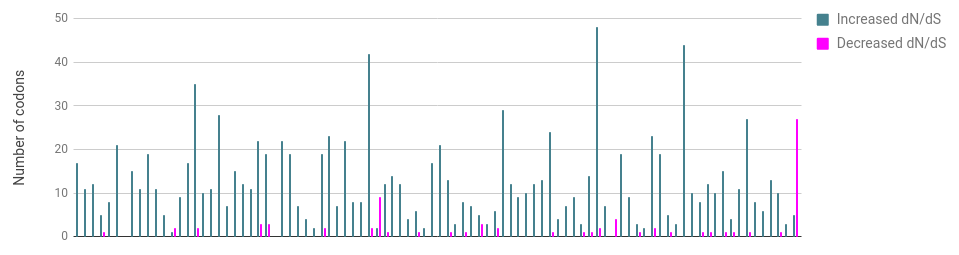
**

**
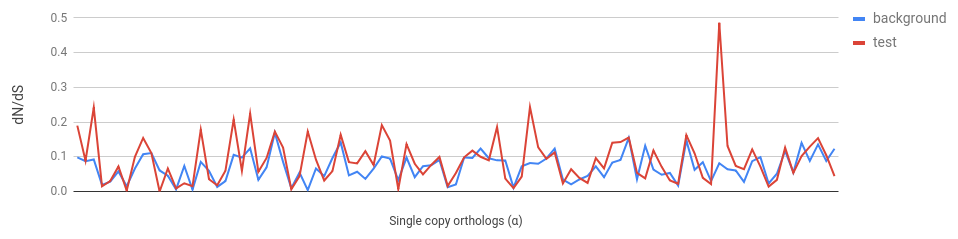
**

**Figure S6.** FEL-contrast analysis results. Up: Sites with change dN/dS in the test branches (mutualistic species) relative to the reference branches (non-mutualistic species) at *p* ≥ 0.05. Down: Global dN/dS estimate based on relative GTR branch lengths and nucleotide substitution biases.
